# Polyphasic taxonomy of green algae strains isolated from Mediterranean freshwaters

**DOI:** 10.1186/s40709-019-0105-y

**Published:** 2019-10-30

**Authors:** Urania Lortou, Spyros Gkelis

**Affiliations:** Department of Botany, Aristotle University of Thessaloniki, P.O. Box 109, 541 24 Thessaloniki, Greece

**Keywords:** Chlorophyta, Chlorophyceae, Freshwater, Molecular systematics, Multi-gene phylogeny, Trebouxiophyceae, Greece

## Abstract

**Background:**

Terrestrial, freshwater and marine green algae constitute the large and morphologically diverse phylum of Chlorophyta, which gave rise to the core chlorophytes. Chlorophyta are abundant and diverse in freshwater environments where sometimes they form nuisance blooms under eutrophication conditions. The phylogenetic relationships among core chlorophyte clades (Chlorodendrophyceae, Ulvophyceae, Trebouxiophyceae and Chlorophyceae), are of particular interest as it is a species-rich phylum with ecological importance worldwide, but are still poorly understood. In the Mediterranean ecoregion, data on molecular characterization of eukaryotic microalgae strains are limited and current knowledge is based on ecological studies of natural populations. In the present study we report the isolation and characterization of 11 green microalgae strains from Greece contributing more information for the taxonomy of Chlorophyta. The study combined morphological and molecular data.

**Results:**

Phylogenetic analysis based on 18S rRNA, internal transcribed spacer (ITS) region and the large subunit of the ribulose-bisphosphate carboxylase (*rbcL*) gene revealed eight taxa. Eleven green algae strains were classified in four orders (Sphaeropleales, Chlorellales, Chlamydomonadales and Chaetophorales) and were represented by four genera; one strain was not assigned to any genus. Most strains (six) were classified to the genus *Desmodesmus*, two strains to genus *Chlorella*, one to genus *Spongiosarcinopsis* and one filamentous strain to genus *Uronema*. One strain is placed in a separate independent branch within the Chlamydomonadales and deserves further research.

**Conclusions:**

Our study reports, for the first time, the presence of *Uronema* in an aquatic environment up to 40 °C and reveals new diversity within the Chlamydomonadales. The results from the ITS region and the *rbcL* gene corroborated those obtained from 18S rRNA without providing further information or resolving the phylogenetic relationships within certain genera, due to the limited number of ITS and *rbcL* sequences available. The comparison of molecular and morphological data showed that they were congruent. Cosmopolitan genera with high worldwide distribution inhabit Greek freshwaters.

## Background

All green algae and embryophyte plants belong to Viridiplantae which were divided early into two evolutionary discrete lineages: the Chlorophyta and Streptophyta [1]. Chlorophyta are an ancient, morphologically and ecologically diverse lineage that include three major classes: Ulvophyceae, Trebouxiophyceae, Chlorophyceae (UTC) [2] and the majority of described species of green algae [1]. Approximately 8000 Chlorophyta species have been described, whilst it is estimated that at least 5000 species still remain undescribed [3]. Despite the fact that the diversity of Chlorophyta is being studied for a long time, our knowledge for their taxonomic and phylogenetic relationships is still deficient [4]. Microscopic green algae are mainly identified based on specific morphological traits (general shape of the cells, position of chloroplasts, presence of pyrenoids, type of reproduction, colony formation, flagella, ultrastructural characteristics etc.) [1]. Nevertheless, morphological identification of microalgae can be very tough due to the absence of obvious structural features in most species and the high degree of variability of several of the observable characteristics within species [5, 6]. The morphological species concept is very subjective, thus the phylogenetic species concept or diagnostic concept [7] has gained a lot of ground [8–10]. As summarized by Krienitz and Bock [11] higher taxonomic lineages such as divisions, classes and orders have been totally revised since molecular phylogenetic methods were introduced into the taxonomy of green algae. Molecular phylogenetic evidence has provided a substantially improved understanding of the relationships among major lineages.

Molecular diversity and phylogeny of isolated members of Chlorophyta derived from Mediterranean freshwaters is a relatively unexplored area; most of the existing studies investigate the ecological status of freshwaters, including morphological identification of microalgae [12–14]. Data on isolation and combined morphological and molecular characterization of Chlorophyta strains are limited and mainly focused on their biotechnological potential [15, 16]. Greece is probably the most diverse Mediterranean country with an excessive level of diversity and endemism of species [17] and it is not rare that new taxa are discovered in animal [18] and microalgal phyla [19, 20]. However, the phylogenetic relationships of Chlorophyta in Greece are barely known as the available information is derived almost exclusively from morphological and/or ecological studies of natural populations [21, 22]. This study investigates the diversity of green algae (Chlorophyta) strains isolated from freshwaters in Greece using a polyphasic approach including morphology, 18S rRNA, ITS, and *rbcL* phylogeny.

## Results

**Fig. 1:**
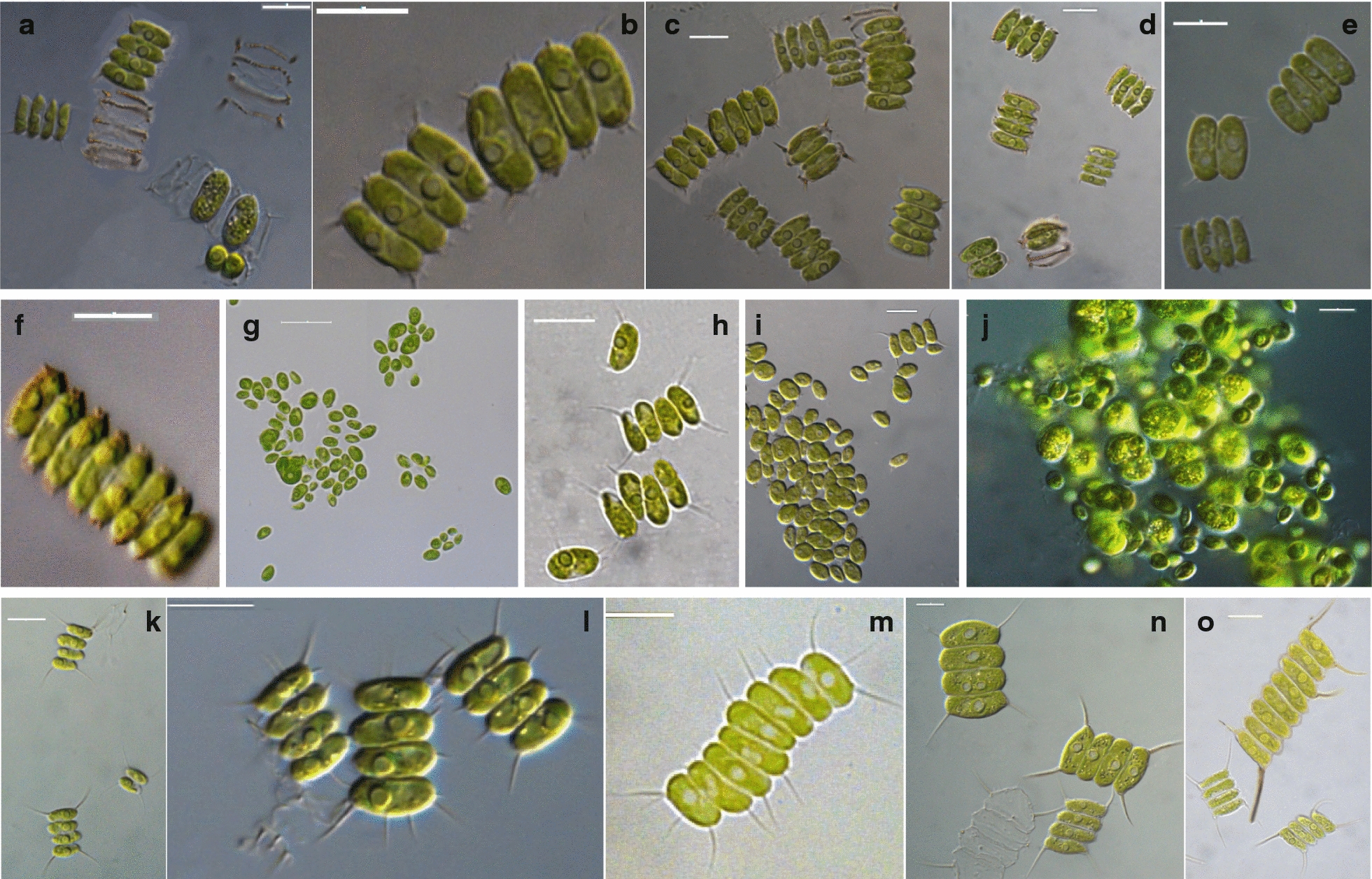
Microphotographs of strains TAU-MAC 0810, 0910, 1010, 2810, 3410 under light microscopy. **a**–**c** *Desmodesmus* sp. TAU-MAC 0910 and **d**–**f** TAU-MAC 1010 demonstrating mature and young coenobia with polar and lateral spines; **a**, **c**, **d** autosporangia, **a**, **d** cell wall ornaments observable from cell wall residues, **f** eight-celled coenobium. **g**–**i** *Desmodesmus abundans* TAU-MAC 0810 and **j**, **k** TAU-MAC 3110; coenobia with polar and lateral spines, unicells and aggregation forms. **l**, **m** *Desmodesmus subspicatus* TAU-MAC 2810; coenobia with lots of lateral spines. **n**, **o** *Desmodesmus communis* TAU-MAC 3410. Bars, 10 μm

**Fig. 2:**
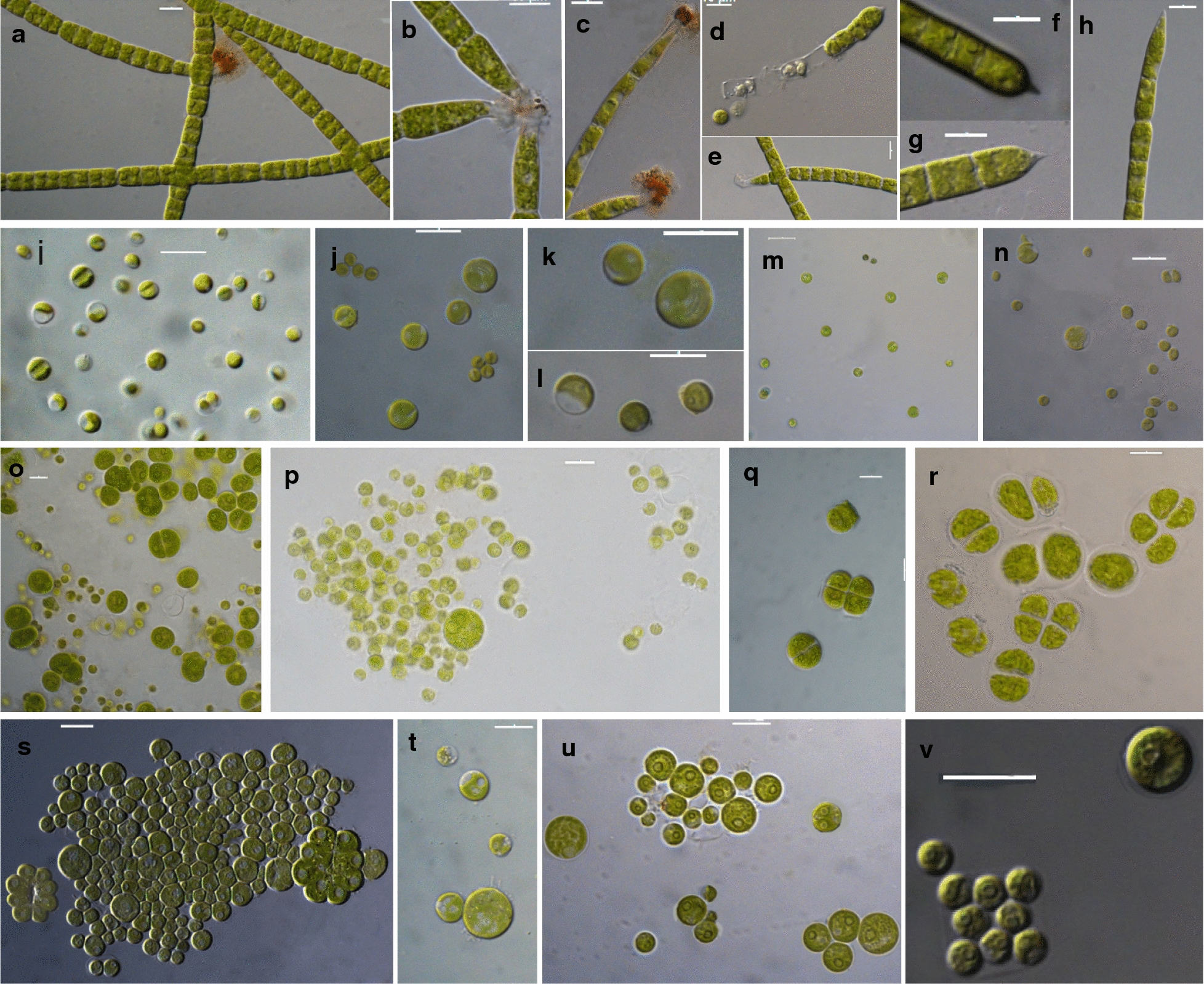
Microphotographs of strains TAU-MAC 0215, 1110, 3210, 3310, 3510 under light microscopy. **a**–**h** *Uronema* sp. TAU-MAC 0215; **a**–**c**, **e** different morphologies of the holdfast, **d** release of aplanospores, **f**–**h** filaments with apical cells showing alterative degree of tapering. **i–k** *Chlorella vulgaris* TAU-MAC 1110 and **l**–**n** TAU-MAC 3210; demonstrating sporangium with two autospores, young and mature cells with one big pyrenoid. **o**–**r** *Spongiosarcinopsis* sp. TAU-MAC 3310; **o**, **p** young vegetative cells derived from zoospores or aplanospores, aplanospores and aplanosporangium, **q**, **r** mature vegetative cells arranged in dyad and tetrad aggregation. **s**–**v** Chlamydomonadales sp. TAU-MAC 3510; **s**–**u** mature vegetative cells form tight aggregations, **v** autospores

**Fig. 3:**
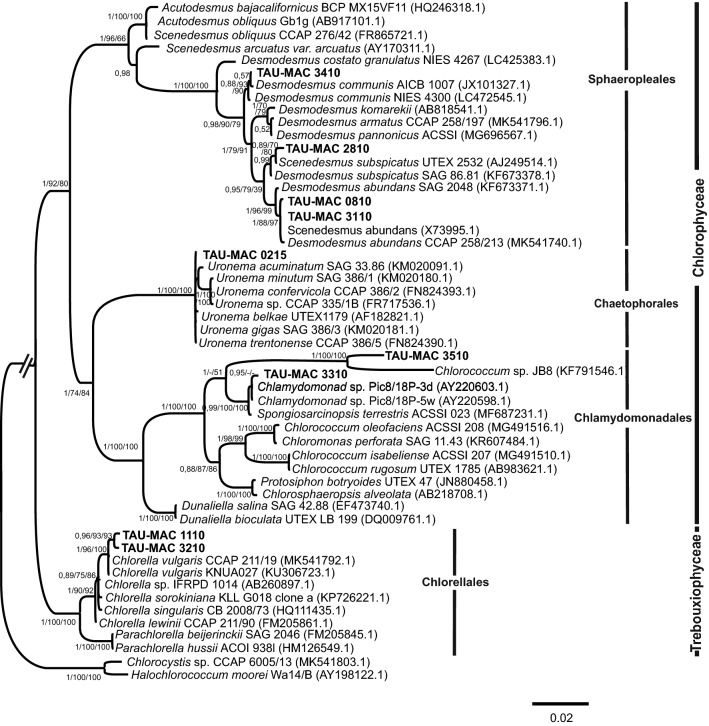
Bayesian inference (BI) phylogenetic tree of relationships of 18S rRNA (c. 1700 bp) of green algae strains isolated from waterbodies of Greece, including the classes Chlorophyceae and Trebouxiophyceae. Support values are indicated as posterior probability for BI and bootstrap support for maximum likelihood (ML) and maximum parsimony (MP) analysis (BI/ML/MP). The numbers in parentheses are GenBank accession numbers

**Fig. 4:**
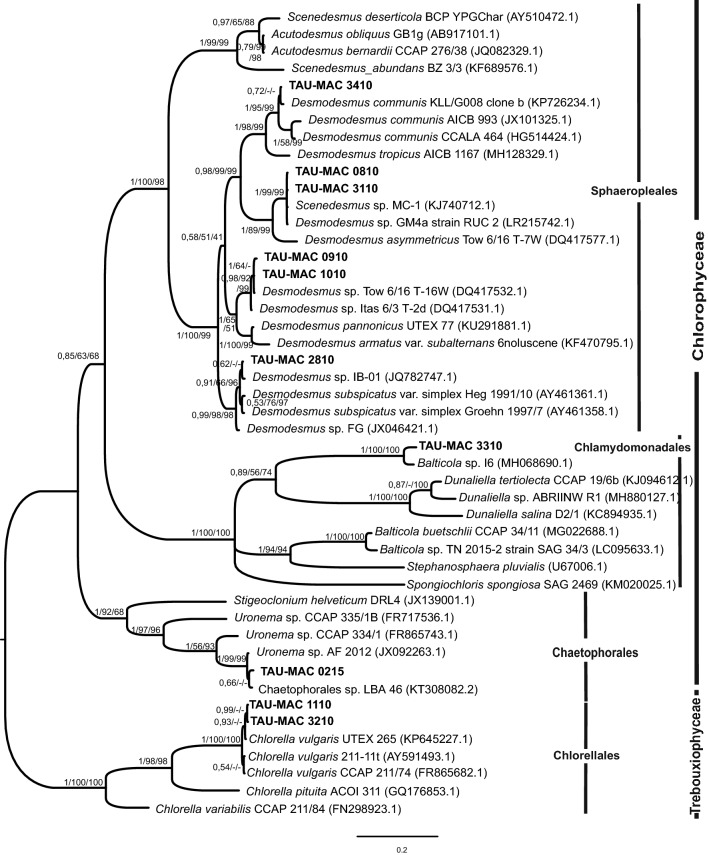
Bayesian inference (BI) phylogenetic tree of relationships of internal transcribed spacer 1 and 2 (ITS1/TS2) rDNA (c. 700 bp) of green algae including the classes Chlorophyceae and Trebouxiophyceae. Support values are indicated as posterior probability for BI and bootstrap support for maximum likelihood (ML) and maximum parsimony (MP) analysis (BI/ML/MP). The numbers in parentheses are GenBank accession numbers

**Fig. 5:**
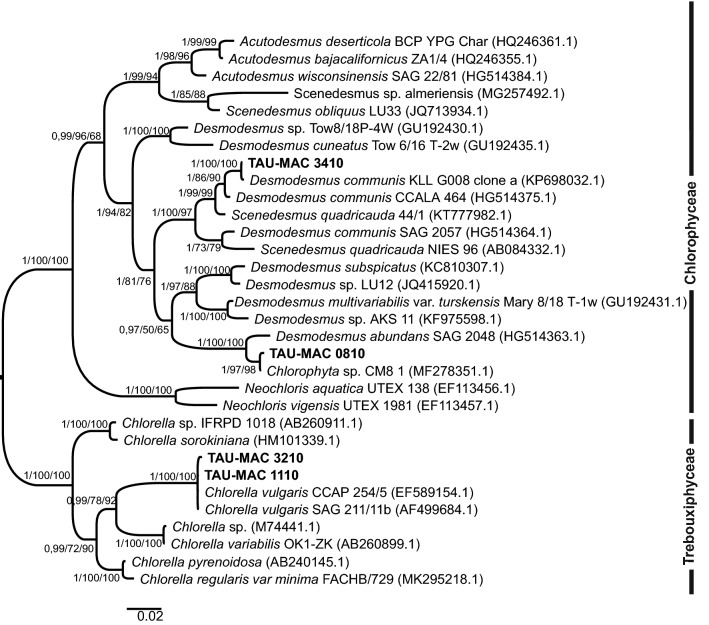
Bayesian inference (BI) phylogenetic tree of relationships of *rbcL* cpDNA (c. 1100 bp) of green algae including the classes Chlorophyceae and Trebouxiophyceae. Support values are indicated as posterior probability for BI and bootstrap support for maximum likelihood (ML) and maximum parsimony (MP) analysis (BI/ML/MP). The numbers in parentheses are GenBank accession numbers

Eleven strains were isolated from surface water samples collected from six lakes (Doirani, Karla, Kastoria, Koronia, Pamvotis, and Volvi) and one hot spring (Agkistro) located in Greece (Table 1). Three morphotypes of Chlorophyta were identified using light microscopy; six strains corresponded to *Desmodesmus*-like species, four strains were coccoid, and one filamentous (Figs. 1, 2). According to morphology (Table 2) and phylogenetic analyses of 18S rRNA (Fig. 3), the 18S–28S ITS region (Fig. 4) and *rbcL* gene (Fig. 5), the isolated strains were placed into clades within the Chlorophyceae and Trebouxiophyceae.

**Table 1:** Green algae strains isolated in this study and their origin

Strain (TAU-MAC)	Origin	Geographic coordinates		Habitat	Collection date
		(N)	(E)		
*Uronema* sp. 0215	Agkistro hot springs	41° 22′ 04″	23° 25′ 40″	Benthic	20/10/2015
*Spongiosarcinopsis* sp. 3310	Lake Doirani	41° 18′ 56″	22° 45′ 37″	Planktic	21/8/2010
*Desmodesmus communis* 3410				Planktic	21/8/2010
*Chlorella vulgaris* 3210	Lake Karla	39° 28′ 29″	22° 51′ 33″	Planktic	10/8/2010
*Desmodesmus abundans* 0810	Lake Kastoria	40° 31′ 11″	21° 15′ 47″	Planktic	26/8/2010
*Desmodesmus* sp. 1010	Lake Koronia	40° 42′ 04″	23° 08′ 17″	Planktic	30/3/2010
*Chlorella vulgaris* 1110				Planktic	15/6/2010
*Desmodesmus abundans* 3110				Planktic	15/6/2010
*Desmodesmus subspicatus* 2810	Lake Pamvotis	39° 40′ 51″	20° 50′ 30″	Planktic	1/11/2010
Chlamydomonadales sp. 3510				Planktic	1/11/2010
*Desmodesmus* sp. 0910	Lake Volvi	40° 40′ 37″	23° 33′ 10″	Planktic	12/7/2010

**Table 2:** Morphometric and morphological characteristics of the TAU-MAC strains isolated from freshwaters in Greece

Strain (TAU-MAC)	Description	Figures	Taxonomic assignment
0910, 1010	Colonial green algae having flat colonies that consist of two or four cells linearly arranged along their long axes. Four-celled coenobia most frequent, but also two-celled and eight-celled were observed. Cell dimensions 8–14 µm long and 3–7 µm wide with one big pyrenoid, short subpolar spines and small lateral. Ribs and rosettes present. Asexual reproduction by aplanosporogenesis	1a–f	*Desmodesmus* sp.
0810, 3110	Cells ovoid and elongated, slightly larger and more elongated in 0810 than in 3110, with one clearly visible pyrenoid surrounded by a sheath of starch plates. The ellipsoidal cells had dimensions 3–9 × 2–6 μm (0810) and 2–7 × 2–5 μm (3110). The single, spineless cells were dominant in 0810 isolate. The rare four-celled coenobia were always spined with two polar and one lateral spine in the outer cells. Two-celled coenobia were the most frequent in 3110 isolate and less commonly four-celled linearly arranged along their long axes. The coenobia had two polar spines in each outer cell, rarely lateral spines were observed. Single cells often had two polar spines. Reproduction by autospores produced by longitudinal division, two to eight per sporangium	1g–k	*Desmodesmus abundans*
2810	Cells ovoid with single large pyrenoid. The two-celled coenobia were common, but four-celled coenobia were predominant and always with lateral spines both in outer and inner cells. Cell dimension 5–13 µm long and 3–7 µm wide. Asexual reproduction by aplanosporogenesis	1l, m	*Desmodesmus subspicatus*
3410	Ellipsoidal cells (11–21 µm long and 3.4–9 µm wide); coenobia linearly arranged with one big pyrenoid per cell covered with starch envelope. The dominant four-celled and rare eight-celled coenobia were always spined with one spine at each pole of outer cells. The entire coenobium was surrounded by an outermost cell wall layer which is visible between poles of inner cells of coenobia. Coenobia with lateral spines in inner cells ware observed very rarely. Asexual reproduction by division of mother cell into 4 daughter cells	1n, o	*Desmodesmus communis*
0215	Filaments unbranched and indefinite in length. Cells are cylindrical, elongated, closely adherent to one another, uninucleate. Cell diameter increases with the age of a filament from 4 to 10 μm and 7 to 23 μm long, containing more than one pyrenoid. Most of the filaments possess a pointed apical cell at the free end which exhibit a different degree of tapering (acuminate, apiculate or attenuate) and a holdfast for attachment by means of a modified basal cell. The morphology of the holdfast also varies from small and colorless to massive and dark brownish-red. The unbranched filaments may undergo fragmentation. Asexual reproduction takes place by aplanosporogenesis or zoosporogenesis	2a–h	*Uronema* sp.
1110, 3210	Cells always spherical and microscopic, 3–9 μm in diameter with one large pyrenoid. Young cells ellipsoidal, becoming spherical at maturity. Reproduction by autosporogenesis usually two autospores per sporangium	2i–n	*Chlorella vulgaris*
3310	Ellipsoidal to spherical solitary young cells 4–9 µm in diameter. Each young cell contains one relatively big nucleus and one pyrenoid covered with starch envelope. Mature cells ovoid to irregular in shape, 19 µm in max. dimension, organized into dyads, tetrads, or packets resulting from desmoschisis. Mature cells remain uninucleate, possessing one to four pyrenoids. Asexual reproduction performed by desmoschisis or zoospores and aplanospores	2o–r	*Spongiosarcinopsis* sp.
3510	Solitary vegetative cells spherical to irregular form, 7–13 μm diameter with the ability to form cell aggregates. One or several pyrenoids, single nucleus or multiple nuclei directly before reproduction by aplanospores. Flagella not visible under the light microscope. Asexual reproduction by aplanospores	2s–v	Chlamydomonadales sp.

Based on 18S rRNA phylogeny (Fig. 3), the strains TAU-MAC 0810, 2810, 3110 and 3410 were clustered together into the Sphaeropleales group, within *Desmodesmus*; strains 2810 and 3410 were placed within *D. subspicatus* and *D. communis*, respectively, whereas the strains 0810, 3110 were clearly positioned within a separate *Desmodesmus* subclade comprised of *D. abundans*. In the order Chaetophorales, strain TAU-MAC 0215 was placed into the *Uronema* clade. Strains TAU-MAC 3510, 3310 and their allied taxa form a separate independent branch within the Chlamydomonadales; 3510 formed a subcluster with a *Chlorococcum* strain and 3310 formed a well-supported (0.99 posterior probability) clade which contains a total of four OTUs: the studied strain, two isolates characterized as “*Chlamydomonad* sp.” (≥ 99.9% similarity) and one strain of the new genus and species *Spongiosarcinopsis terrestris*. A direct comparison of sequence similarities among the strains 1110, 3210 and *Chlorella vulgaris* showed a 99% sequence identity. Nevertheless they formed a distinct subclade inside *Chlorella vulgaris* clade (Fig. 3). The well-supported relationships for strains TAU-MAC 0810, 1110, 2810, 3110, 3210, 3410, 0215 identified in the 18S rRNA analysis were also found in the ITS phylogeny (Fig. 4). Strains 0910, 1010 formed a clade with unidentified *Desmodesmus* species. The closest relative of strain 3310 based on the ITS region, with 96% identity, was a *Balticola* (MH068690.1) sequence. Sequences from *rbcL* regions were successfully amplified and sequenced only for fours strains: TAU-MAC 0810, 1110, 3210, and 3410. Molecular data based on *rbcL* just corroborated the results coming from 18S rRNA and ITS phylogenies: strain 0810 formed a subclade with an unidentified species within *D*. *abundans* clade, 3410 clustered within *D*. *communis* clade and isolates 1110, 3210 clustered together with *Chlorella vulgaris* sequences (Fig. 5). The different tree construction methods produced similar trees.

The morphology of *Desmodesmus*-like strains corresponded to *Desmodesmus* as all of them had spines, in contrast to *Scenedesmus* spp. which have a smooth, non-ornamented cell wall. The morphological and morphometric characteristics of each strain are given in Table 2. Under light microscopy coenobia of the strains TAU-MAC 0810 and 3110 were always spined with two polar and one lateral spine in the outer cells (Fig. 1g–k), a common trait of *Desmodesmus abundans.* The morphology of the strain TAU-MAC 2810 was similar to *Desmodesmus subspicatus*; coenobia were four-celled and always with lots of spines both in outer and inner cells (Fig. 1l, m). Strain TAU-MAC 3410 matched the morphological structure of *Desmodesmus communis*; four-celled coenobia bearing four corner spines on the end cells of colonies (Fig. 1l, m). Strains TAU-MAC 0910 and 1010 exhibited intermediate structures to *D. abundans* and *D. communis* but with shorter subpolar and lateral spines and were classified as *Desmodesmus* sp. (Fig. 1a–f). Under light microscopy, strains TAU-MAC 1110 and 3210 were the classical “green balls”, tiny, solitary-living and simply propagating spherical microalgae (Fig. 2i–n), matching the morphological description of *Chlorella vulgaris.* Coenobia of the strain TAU-MAC 3310 were organized into dyads, tetrads, or packets resulting from desmoschisis (Fig. 2o–r), as in the recently described genus *Spongiosarcinopsis*; species discrimination was not possible due to lack of data. The strain TAU-MAC 3510 exhibited solitary, spherical cells with the ability to form cell aggregates (Fig. 2s–v) but no other characteristic traits or flagella were observed, therefore we could not assign it to any genus of the Chlamydomonadales. Under light microscopy the strain TAU-MAC 0215 displayed morphological traits of the genus *Uronema*; filaments uniseriate, unbranched, attached to substratum by holdfast, bearing a pointed apical cell at the free end (Fig. 2a–h). For most of the strains, the comparison of molecular and morphological data showed that they were congruent. Interestingly, both morphology and phylogeny placed the strain TAU-MAC 3510 within the Chlamydomonadales, but provided insufficient evidence to identify a genus.

## Discussion

In this study we report for the first time the isolation and polyphasic taxonomy of freshwater green algae strains from Greece belonging to the genera *Desmodesmus*, *Chlorella*, *Spongiosarcinopsis* and *Uronema*. A small number of *Tetraselmis* strains have been previously isolated from Greek lagoons [23] but freshwater species have been characterized mainly through microscopic observations [22, 24–27] or molecular cloning of the 18S rRNA gene [28, 29]. Most of the TAU-MAC strains (0810, 0910, 1010, 2810, 3110 and 3410) belong to the genus *Desmodesmus*, one of the most abundant genera in fresh to brackish waters all over the globe [30]. Their cosmopolitan occurrence illustrates the wide range of environmental conditions these organisms can tolerate due to the high degree of polymorphism they exhibit [31, 32]. The genus *Desmodesmus* was separated from *Scenedesmus* sensu lato based on ITS2 sequences [33] but *Desmodesmus* species taxonomy remains one of the most long-standing issues in green microalgal systematics [34]. Many of the *Desmodesmus* strains/species display considerable morphological variability due to nutrient availability, environmental signals and culture conditions [34, 35]. This issue has led to the description of more than 1300 species and sub-specific taxa in the former genus *Scenedesmus*, of which many are invalid [36]. This is obvious from our phylogenetic trees where, within *Desmodesmus* clades, not revised sequences previously characterized as *Scenedesmus* are included (e.g. the sequences AJ249514.1 and X73995.1 which are described as *Scenedesmus* but actually refer to *D. subspicatus* and *D. abundans*, respectively). Strains TAU-MAC 0810 and 3110 were identified as *Desmodesmus abundans* by molecular and morphological analysis. Because of the extensive morphological variability of the species [37], organisms previously belonging even in different classes (e.g. *Chlorella fusca* SHIH. et KRAUSS) have been reassessed as *D. abundans* [37, 38]. Different morphological structures were observed in our cultures also. Molecular phylogenies based on the 18S rRNA and ITS regions placed strain 2810, with very high node support, in a clade with *Desmodesmus subspicatus* strains isolated from different freshwaters worldwide [38–42]. However, this strain was isolated from the hypertrophic, shallow Lake Pamvotis where heavy toxic cyanobacterial blooms are frequently formed [43]. Morphological taxonomy was congruent to the molecular classification [35]. Strains TAU-MAC 0910 and 1010 were identified as *Desmodesmus* as well, since they plainly clustered together with unidentified species of the genus. Phylogenetic analyses could not be conclusive down to the species level as their DNA was amplified only with ITS region primers. Moreover, morphology did not allow species delimitation, owing to morphological criteria we could not point out, due to the phenotypic plasticity of *Desmodesmus* and the similarity of structures between *Desmodesmus* species [31, 44]. In the 18S rRNA and ITS phylogeny all sequences that clustered together into well-supported clades with the strain TAU-MAC 3410 are *Desmodesmus communis* [also known with the traditional name *Scenedesmus quadricauda* Turp. (Bréb)], isolated from Europe to Asia [45, 46]. The alignment of these sequences with 3410 showed identity up to 99%. The *rbcL*-based phylogeny supports the previous results, since 3410 clustered together with a *Scenedesmus quadricauda* sequence [47] probably belonging to a non-revised *D. communis* [45]. Under light microscopy, TAU-MAC 3410 displayed the common structure of the species [46, 48]. The species has a wide distribution in freshwaters, mainly in those with moderate temperature and in the slightly eutrophicated ones [46] but our strain was isolated from Lake Doirani, an extremely eutrophic lake [49]. Species of the genus have been identified in freshwaters of Greece [21, 25, 27, 50].

Greek lakes also host *Chlorella vulgaris*, the archetypical form of coccoid green algae firstly described by Beijerinck in 1890 [51]. Molecular phylogeny based on three molecular markers placed strains TAU-MAC 1110 and 3210, with high bootstrap values, in clades with *Chlorella vulgaris* strains isolated from various environments. The microscopic observations merely confirmed the molecular results [51, 52]. *Chlorella* and its relatives belong to the most common aquatic, terrestrial and aerophytic algae with ubiquitous distribution [53]. The sequences clustering together with TAU-MAC strains originate from different habitats of USA, Europe, New Zealand and Japan, while TAU-MAC strains were isolated from the two hypertrophic Lakes Pamvotis and Koronia [22, 25, 54]. This is the first report of *Chlorella vulgaris* presence in these lakes. Furthermore, because of its tiny size, population size and physiological ability to tolerate desiccation or other types of abiotic stress, *Chlorella* has been reported as an air-dispersed microorganism in Greece [24, 55].

The phylogenetic position with maximal bootstrap value of TAU-MAC 0215 isolated from hot springs clustered with representatives of the filamentous genus *Uronema*. Under light microscopy, the strain exhibited the typical morphological features of the genus [56, 57]. Its 18S rRNA sequence showed 99–100% pairwise sequence identity with all *Uronema* species included in the clade, whilst in ITS phylogeny the closest relative (98.2% similarity) was an unidentified *Uronema* sequence. Representatives of the genus appear as benthic algae in mesotrophic and eutrophic ecosystems while are common in both terrestrial and aquatic environments [56, 58]. The strains clustering together with TAU-MAC 0215 have a wide distribution since they are derived from North America to Europe. To the best of our knowledge, this is the first report of the presence of *Uronema* in an aquatic environment up to 40 °C. Representatives of *Uronema* in Greece have been previously reported in Lake Amvrakia [59].

The strains TAU-MAC 3310 and 3510 were placed in a separate independent branch within the Chlamydomonadales, the largest group of Chlorophyceae with a complex taxonomic history [1]. Phylogenetic analyses of nuclear and chloroplast DNA sequences have changed the concept of the class: this clade includes a large number of Chlamydomonadales taxa, plus taxa formerly placed in Dunaliellales, Chlorococcales, Tetrasporales, Chlorosarcinales, Volvocales, and Chaetophorales as summarized by Leliaert et al. [1]. The closest 18S rRNA sequence to TAU-MAC 3510 was the strain *Chlorococcum* sp. JB8 isolated from extreme saline–alkali soil in China [60]; however, the low pairwise sequence similarity (96%) and the lack of other morphological traits suggest that our strain forms a novel clade within Chlamydomonadales. The closest relative of TAU-MAC 3310 with 99% similarity (18S rRNA) was a strain isolated from a gray forest soil in Russia belonging to the novel, recently described genus and species *Spongiosarcinopsis terrestris* [61], together with two unclassified representatives of Chlamydomonadales (Pic8/18P-3d and Pic8/18P-5w) derived from an eutrophic pond in Itaska State Park, Minnesota [62]. In 18S rRNA phylogeny, 3310 was placed in a well-supported clade with the two isolates mentioned previously, forming a distinct subclade inside the *Spongiosarcinopsis* clade. Even if the strain displayed similar morphological characteristics to *S. terrestris*, the different niche (aquatic vs. terrestrial) and the separate subclade suggest that our strain together with Pic8/18P-3d and Pic8/18P-5w belong to a different *Spongiosarcinopsis* species and deserve further research. Buchheim et al. [63] studying the phylogeny of the Chlamydomonadales, compared nuclear-encoded small-subunit rRNA sequences and chloroplast-encoded large subunit rRNA sequences from flagellate green algae. Their analyses showed that the chloroplast phylogenies are clearly more robust than the nuclear phylogenies suggesting that the chloroplast data are more variable having a greater density of genetically informative sites than the nuclear data. Unfortunately, the *rbcL* gene could not be amplified in strains 3510 and 3310. Studies discuss the universality of primers for this marker and they indicate the need to design more efficient and robust primers for a broader coverage of plant species [64, 65].

## Conclusions

Polyphasic taxonomy based on three genetic markers (18S rRNA, ITS and *rbcL* gene) and morphology divulged eight taxa among eleven strains from Greek freshwaters. Most of the strains were identified as *Desmodesmus.* Some representatives of the genus have already been recognized in Greek freshwaters but this is the first time they are isolated and identified by molecular analyses. Two strains could be assigned to *Chlorella vulgaris* and this is the first report of its presence in the lakes where they were isolated from. One filamentous strain belongs to the genus *Uronema*, reported for the first time in an aquatic environment up to 40 °C. Two strains (3310, 3510) are placed in a separate independent branch within the Chlamydomonadales indicating novel diversity that deserves further investigation. Green algae isolated from Greek lakes seem to be cosmopolitan and non-toxic. Molecular data from the three genetic markers converged but more submitted sequences are needed to clarify relationships within the genera.

## Methods

### Sample collection, isolation and culture

Strains were isolated from water samples collected from six freshwaters of Greece and from an algal mat in a thermal spring between 2010 and 2015 (Table 1). Water samples were collected from the surface layer (0–0.5 m) of inshore sites, details are given in Gkelis and Zaoutsos [66]. The algal mat sample was collected after carefully scratching with a sterile scalpel the mat and placing the detached mat in sterile 50 mL polyethylene vessels. All sampling sites were chosen based on existing data on the occurrence of green algae [21, 29, 50, 67] or in situ observations. For a description of the lakes, see [22, 68, 69]. Thermal springs of Agkistro are located in the homonymous village, the northernmost village of Serres (N. Greece) and they feed the area’s thermal spa facilities, dating back to 925 AD [70]. The hot spring water (40 °C) is characterized as meteoric, sterile, oligometallic, sodium, calcium, sulphate, oxycarbonate, potassium, fluoride, hypotonic, thermal water [71, 72]. Strains were isolated on solid growth media using classical microbiological techniques and grown as batch clonal unialgal cultures [73]. The algal strains are deposited in Aristotle University of Thessaloniki MicroAlgae and Cyanobacteria Culture Collection (TAU-MAC) [74] and can be accessed at [75].

Strains were cultured in BG-11 medium with nitrogen [76] and maintained in the same medium by regular subculturing every 2 weeks. Cultures were grown as liquid batch cultures at a photosynthetic photon flux density of 20 μmol m^−2^s^−1^ using cool white light fluorescent tubes (Sylvania Standard F36W/154-T8, SLI) at 20 ± 2 °C in culture room, in a 16:8 h light:dark cycle.

### Light microscopy and morphology

A Zeiss Axio Ιmager.Z2 (Carl Zeiss, Germany) microscope using bright field and differential interference contrast was used. Microphotographs were taken with an Axio Cam MRc5 digital camera (Carl Zeiss, Germany). Strains were identified using special taxonomic papers [35, 37, 38, 46, 48, 51, 52, 56, 57, 61]. Mean cell dimensions were calculated after measuring the dimension of at least 50 cells of each strain.

### DNA extraction and PCR

The protocol described in Atashpaz et al. [77] was used to extract DNA from algae. PCR was carried out using the primer pairs and under the conditions described in Table 3. Thermal cycling was carried out using an Eppendorf MasterCycler Pro (Eppendorf). The PCR products were visualized on a 1.2% w/v agarose gel in 1× TAE buffer under UV light, and were purified using the Nucleospin^®^ Gel and PCR Clean-up kit (MACHEREY-NAGEL).

**Table 3:** PCR primers used in the analyses of green-algae strains isolated from freshwaters of Greece

Primer	Target gene-region	Sequence (5′–3′)	Size (bp)	References	Conditions
EukA	18S rRNA	AACCTGGTTGATCCTGCCAGT	1750	[78]	Initial denaturation step at 95 °C for 5 min, 35 cycles consisting of denaturation at 95 °C for 60 s, annealing at 55 °C for 60 s and elongation at 72 °C for 90 s; a final 7-min elongation step at 72 °C was included
EukB		TGATCCTTCTGCAGGTTCACCTAC			
U1391R		GGGCGGTGTGTACAARGR		[79]	
ITS-AF	ITS	CGTTTCCGTAGGTGAACCTGC	700	[80]	Initial denaturation step at 94 °C for 4 min, 35 cycles consisting of denaturation at 95 °C for 60 s, annealing at 58 °C for 2 min and elongation at 72 °C for 2 min; a final 7-min elongation step at 72 °C was included
ITS-BR		CATATGCTTAAGTTCAGCGGG T			
*rbcL*1-20	*rbcL*	ATGGTTCCACAAACAGAAAC	1100	[81]	
*rbcL*1181-1160		AAGATTTCAACTAAAGCTGGCA			

### Analysis of sequence data

For each individual strain, forward and reverse reads were assembled and the assembled sequences were checked for chimeras using the RDPII chimera detection [82]. Sequence data were visually inspected using BioEdit (Ibis Biosciences 1997–2015^©^) and the sequences were edited manually, where necessary. For the detection of closest relatives, all sequences were compared with the BLAST function [83] and aligned with sequences obtained from GenBank [84] databases, using the ClustalW [85] alignment utility through MEGA6 software [86]. Phylogenetic analyses were performed using maximum parsimony (MP) and maximum likelihood (ML) methods implemented in MEGA6 and the confidence of the tree topologies was checked using bootstrap analyses (1000 replicates). Using the jModelTest 0.1.1 [87], the GTR+I+G model was determined as the most appropriate and was used for all ML and BI analyses (18S rRNA, ITS, *rbcL*). Bayesian phylogenetic analyses were also performed using MrBayes 3.2.1 [88] with 10,000,000 generations of Markov chain Monte Carlo iterations (MCMC), discarding the first 25% as burn-in and the following datasets were sampled every 1000th generation. The nucleotide sequences of the partial 18S rRNA, ITS and *rbcL* gene regions from chlorophyta strains in this study were deposited in GenBank with accession numbers MK496891–MK496899, MK496922–MK496931 and MK503332–MK503335, respectively.

## Data Availability

The algal strains are deposited in Aristotle University of Thessaloniki MicroAlgae and Cyanobacteria Culture Collection (TAU-MAC) and can be accessed at [75]. The datasets used and/or analyzed are included in this manuscript.
